# Correction: Effector-mediated subversion of proteasome activator (PA)28αβ enhances host defense against *Legionella pneumophila* under inflammatory and oxidative stress conditions

**DOI:** 10.1371/journal.ppat.1011604

**Published:** 2023-08-22

**Authors:** Tshegofatso Ngwaga, Deepika Chauhan, Abigail G. Salberg, Stephanie R. Shames

The file for S1 Fig is incorrect in the published article. The correct S1 Fig can be viewed below.

## Supporting information

S1 Fig TNF secretion from L. pneumophila-infected BMDMs is not increased by LegC4 or loss of PA28αβ. TNF WT or *Psme1/2*^*-/-*^ BMDMs infected for **(A)** 8 h or **(B)** 24 h with *L*. *pneumophila ΔflaA*, *ΔflaAΔlegC4* (pEV), *ΔflaAΔlegC4 (plegC4)*, or the avirulent *ΔdotA* control at a multiplicity of infection of 10. Plasmid expression of *legC4* was induced with 1 mM IPTG. Data shown are mean ± s.d. on samples in triplicates for a single experiment and are representative of results from three independent experiments. Asterisks denote statistical significance by two-way ANOVA (*P<0.05; **P<0.05). ns; not significant.
(TIF)
