# Supplementary figures and images for: Effector-mediated subversion of proteasome activator (PA)28αβ enhances host defense against Legionella pneumophila under inflammatory and oxidative stress conditions
Source: PLoS Pathog. 2023 Jun 22;19(6):e1011473. doi: 10.1371/journal.ppat.1011473 (PMC10321654; doi:10.1371/journal.ppat.1011473)

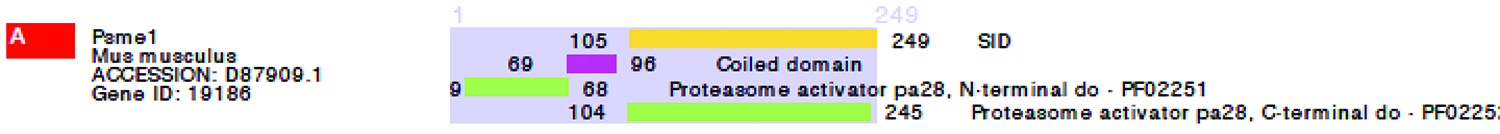

Supplement: S1 Fig — Schematic diagram of yeast two-hybrid results showing mouse PA28α (Psme1) domains and the selected interaction domain (SID) from amino acid residues 105–249 contained in all clones shown to interact with LegC4. (TIF) [file ppat.1011473.s001.tif]

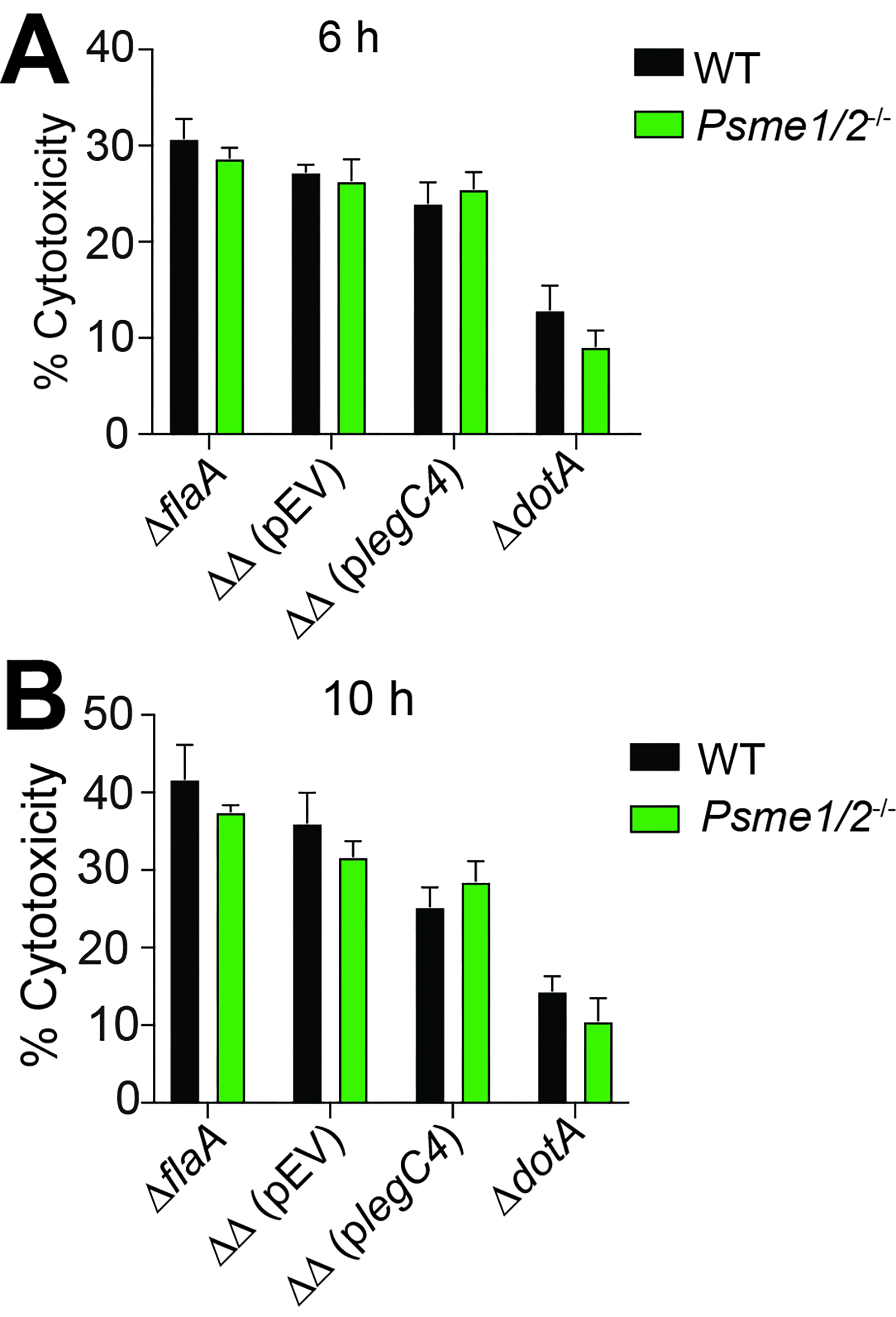

Supplement: S2 Fig — TNF WT or Psme1/2-/- BMDMs infected for (A) 8 h or (B) 24 h with L. pneumophila ΔflaA, ΔflaAΔlegC4 (pEV), ΔflaAΔlegC4 (plegC4), or the avirulent ΔdotA control at a multiplicity of infection of 10. Plasmid expression of legC4 was induced with 1 mM IPTG. Data shown are mean ± s.d. on samples in triplicates for a single experiment and are representative of results from three independent experiments. Asterisks denote statistical significance by two-way ANOVA (*P<0.05; **P<0.05). ns; not significant. (TIF) [file ppat.1011473.s002.tif]

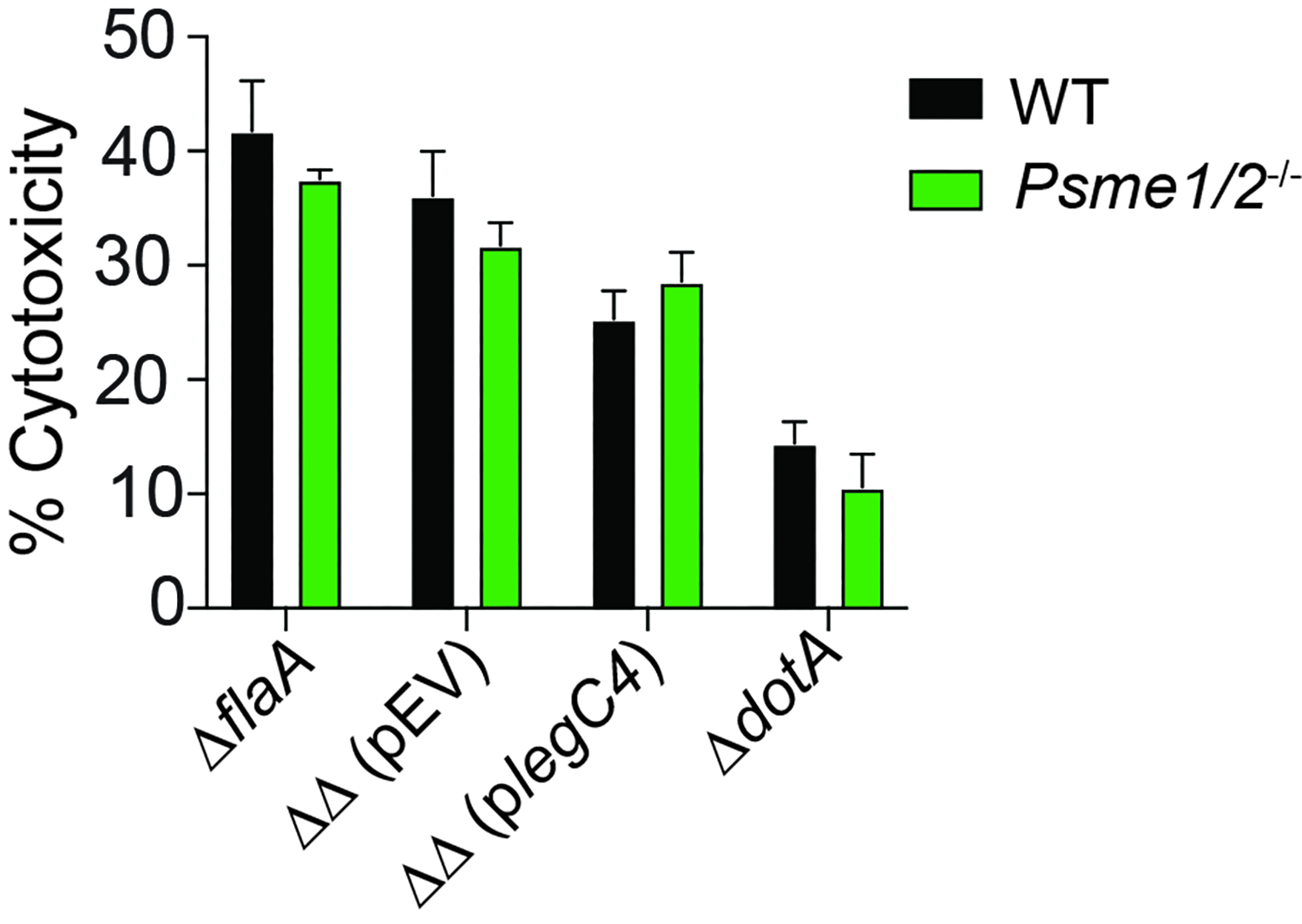

Supplement: S3 Fig — WT or Psme1/2-/- BMDMs were infected in triplicates with L. pneumophila strains (MOI of 10) and LDH in cell supernatants was quantified at 10h post-infection. Percent cytotoxicity was calculated by normalizing absorbance values to a lysis control (100% cytotoxicity). UI, uninfected cells. Plasmid expression of legC4 was induced with 1 mM IPTG. Data shown are mean ± s.d. of triplicate samples for a single experiment and are representative of results from three independent experiments. (TIF) [file ppat.1011473.s003.tif]

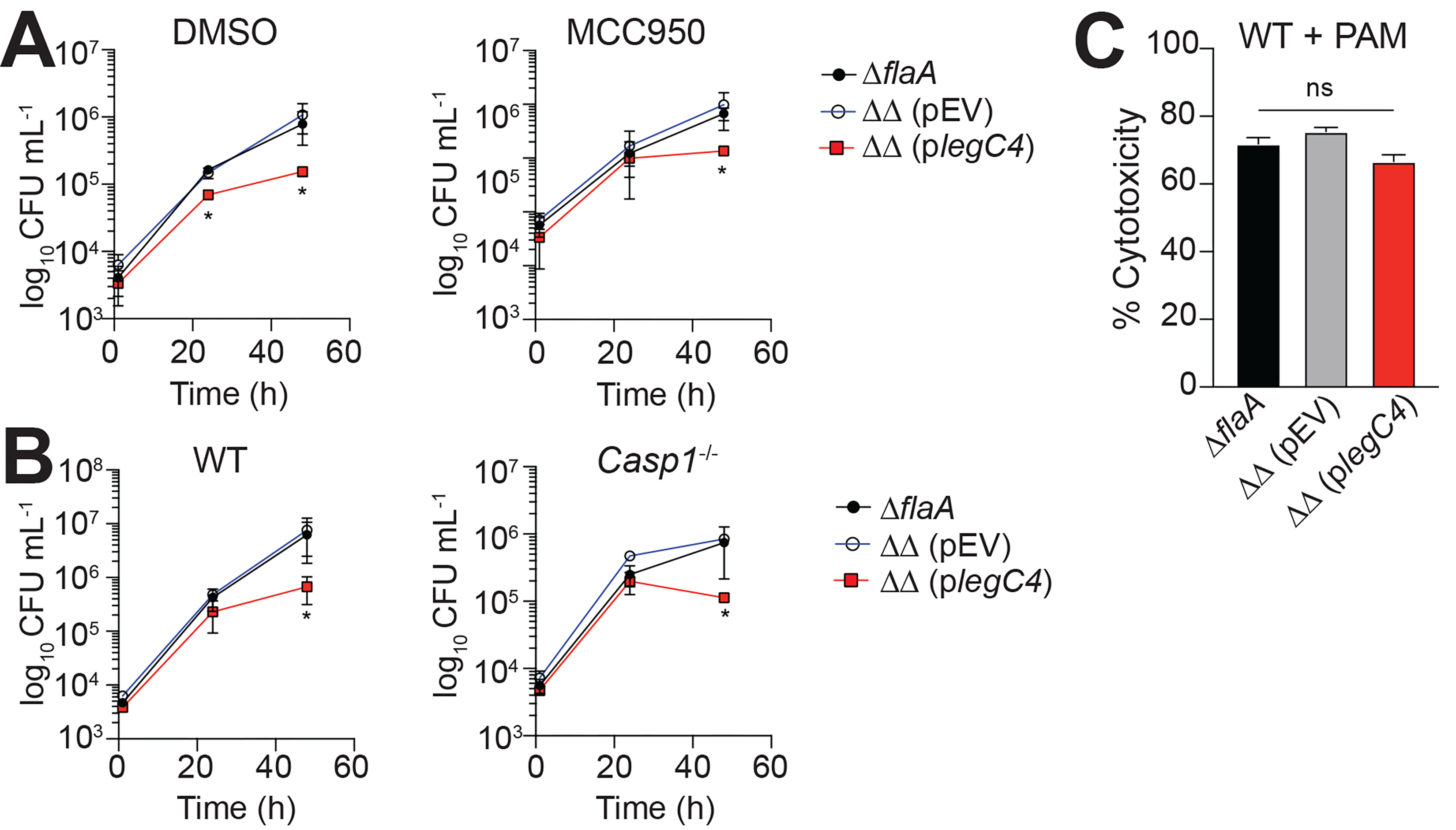

Supplement: S4 Fig — (A) WT BMDMs were infected with L. pneumophila strains (MOI of 1) in the presence of either 1μM MCC950 or volume equivalent of DMSO (vehicle) and CFU were enumerated at the indicated time points. (B) WT and Casp1-/- BMDMs were infected with L. pneumophila (MOI of 1) and CFU were enumerated at the indicated time points. Data are shown as mean ± s.d. of pooled results of two independent experiments with triplicate wells per condition in each experiment. Asterisks denote statistical significance by Two-way ANOVA (*P<0.05; **P<0.01). Plasmid expression of legC4 was induced with 1 mM IPTG. (C) WT BMDMs were primed with 1 μM PAM3CSK4 (PAM) for 24 h and infected with the indicated L. pneumophila strains at an MOI of 10 for 6 h and cytotoxicity was quantified by LDH release assay. Data shown are mean ± s.d. of triplicate samples for a single experiment and are representative of results from three independent experiments. Ns; not significant by Two-way ANOVA. (TIF) [file ppat.1011473.s004.tif]

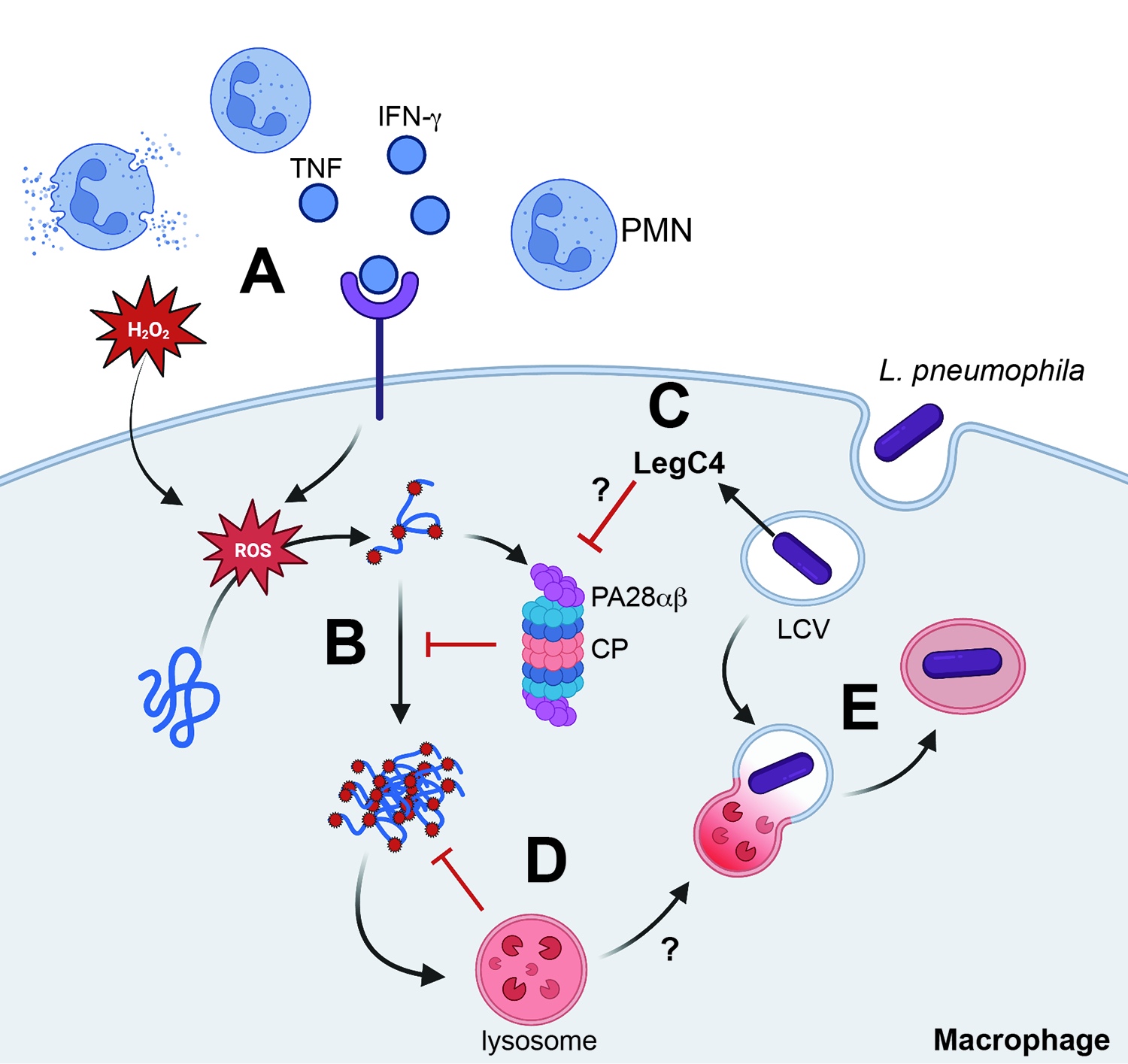

Supplement: S5 Fig — (A) TNF, IFN-γ and reactive oxygen species (ROS) are produced by infected and bystander immune cells induce oxidative stress in activated macrophages via intracellular ROS production and by passive diffusion of membrane-permeable extracellular ROS (H2O2). (B) ROS indiscriminately carbonylate amino acid side chains, which perturbs protein folding and function, and turnover of these damaged proteins is mediated by PA28αβ-CP proteasomes. (C) LegC4 translocated into L. pneumophila-infected macrophages binds PA28α and modulates its activity by an unknown mechanism. (D) Impaired proteasome activity leads to formation of carbonylated protein aggregates, which are impervious to proteasomal degradation and trigger upregulation of lysosome biogenesis and fusogenic activity. (E) Increased phagolysosomal fusion with the Legionella-containing vacuole (LCV) may result from global increases in lysosomal degradation to maintain proteostasis under oxidative stress conditions. Image created with Biorender.com. (TIF) [file ppat.1011473.s005.tif]
