# Supplementary material for: Effector-mediated subversion of proteasome activator (PA)28αβ enhances host defense against Legionella pneumophila under inflammatory and oxidative stress conditions
Source: PLoS Pathog. 2023 Jun 22;19(6):e1011473. doi: 10.1371/journal.ppat.1011473 (PMC10321654; doi:10.1371/journal.ppat.1011473)
Supplement: S1 Text — The supplemental text contains a list of Legionella strains (Table A), plasmids (Table B), and oligonucleotide primers (Table C) used in this study, and corresponding references. (DOCX) [file ppat.1011473.s007.docx]

**Supplementary Information (SI)**

**SI Figures**


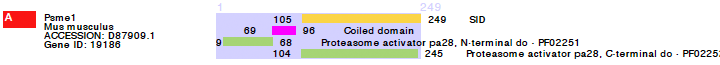


**Figure S1. The C-terminal domain of PA28α was present in all clones recovered by Y2H.** Schematic diagram of yeast two-hybrid results showing mouse PA28α (*Psme1*) domains and the selected interaction domain (SID) from amino acid residues 105-249 contained in all clones shown to interact with LegC4.

**Figure S2.** **TNF secretion from *L. pneumophila*-infected BMDMs is not increased by LegC4 or loss of PA28αβ.** TNF WT or *Psme1/2*^-/-^ BMDMs infected for **(A)** 8 h or **(B)** 24 h with *L. pneumophila* ∆*flaA*, ∆*flaA*∆*legC4* (pEV), ∆*flaA*∆*legC4* (p*legC4*), or the avirulent ∆*dotA* control at a multiplicity of infection of 10. Plasmid expression of *legC4* was induced with 1 mM IPTG. Data shown are mean ± s.d. on samples in triplicates for a single experiment and are representative of results from three independent experiments. Asterisks denote statistical significance by two-way ANOVA (**P*<0.05; ***P*<0.05). ns; not significant.

.

**Figure S3. Neither LegC4 nor loss of PA28αβ impair viability of *L. pneumophila*-infected BMDMs.** WT or *Psme1/2*-/- BMDMs were infected in triplicates with *L. pneumophila* strains (MOI of 10) and LDH in cell supernatants was quantified at 10h post-infection. Percent cytotoxicity was calculated by normalizing absorbance values to a lysis control (100% cytotoxicity). UI, uninfected cells. Plasmid expression of *legC4* was induced with 1 mM IPTG. Data shown are mean ± s.d. of triplicate samples for a single experiment and are representative of results from three independent experiments.

**Figure S4. LegC4-mediated restriction is not dependent on caspase-1 or NLRP3 inflammasome activity**. **(A)** WT BMDMs were infected with *L. pneumophila* strains (MOI of 1) in the presence of either 1µM MCC950 or volume equivalent of DMSO (vehicle) and CFU were enumerated at the indicated time points. **(B)** WT and *Casp1*^-/-^ BMDMs were infected with *L. pneumophila* (MOI of 1) and CFU were enumerated at the indicated time points. Data are shown as mean ± s.d. of pooled results of two independent experiments with triplicate wells per condition in each experiment. Asterisks denote statistical significance by Two-way ANOVA (**P*<0.05; ***P*<0.01). Plasmid expression of *legC4* was induced with 1 mM IPTG. **(C)** WT BMDMs were primed with 1 µM PAM_3_CSK_4_ (PAM) for 24 h and infected with the indicated *L. pneumophila* strains at an MOI of 10 for 6 h and cytotoxicity was quantified by LDH release assay. Data shown are mean ± s.d. of triplicate samples for a single experiment and are representative of results from three independent experiments. Ns; not significant by Two-way ANOVA.

**Figure S5.** Schematic model of the LegC4 restriction mechanism. **(A)** TNF, IFN-γ and reactive oxygen species (ROS) are produced by infected and bystander immune cells induce oxidative stress in activated macrophages via intracellular ROS production and by passive diffusion of membrane-permeable extracellular ROS (H_2_O_2_). **(B)** ROS indiscriminately carbonylate amino acid side chains, which perturbs protein folding and function, and turnover of these damaged proteins is mediated by PA28αβ-CP proteasomes. **(C)** LegC4 translocated into *L. pneumophila*-infected macrophages binds PA28α and modulates its activity by an unknown mechanism. **(D)** Impaired proteasome activity leads to formation of carbonylated protein aggregates, which are impervious to proteasomal degradation and trigger upregulation of lysosome biogenesis and fusogenic activity. **(E)** Increased phagolysosomal fusion with the *Legionella*-containing vacuole (LCV) may result from global increases in lysosomal degradation to maintain proteostasis under oxidative stress conditions. Image created with Biorender.com.

**SI Text**

**Table A.** *Legionella* strains used in this study

| **Strain** | **Description** | **Resistance** | **Ref(s)** |
| --- | --- | --- | --- |
| *Legionella pneumophila* SRS43 | | | |
| ∆*flaA* | FlaA-deficient parental strain | Sm^R^ | [1] |
| ∆*flaA*∆*legC4* | LegC4-deficient ∆*flaA* strain | Sm^R^ | [1] |
| ∆*flaA*∆*legC4* (pEV) | Harboring empty pSN85 vector | Sm^R^, Cm^R^ | [1] |
| ∆*flaA*∆*legC4* (p*legC4*)^a^ | Harboring pSN85::*legC4* | Sm^R^, Cm^R^ | [1] |
| ∆*flaA*∆*legC4* (pJB) | Harboring empty pJB1806 vector | Sm^R^, Cm^R^ | [1] |
| ∆*flaA*∆*legC4* (pJB*legC4*)^b^ | Harboring pJB1806::p*legC4* | Sm^R^, Cm^R^ | [1] |
| ∆*dotA* | Avirulent control | Sm^R^, Cm^R^ | [2] |

a – gene expression induced with 1 mM IPTG

b – *legC4* expression from endogenous promoter

**Table B.** Plasmids used in this study

| **Plasmid** | **Description** | **Resistance** | **Ref(s)** |
| --- | --- | --- | --- |
| *L. pneumophila* expression | | | |
| pSN85 (pEV) | *L. pneumophila* expression vector | Cm^R^ | [3] |
| pSN85::*legC4* (p*legC4*)^a^ | For expression of *3xflag-legC4* | Cm^R^ | [1] |
| pJB1806 (pJB) | *L. pneumophila* expression vector | Cm^R^ | [4] |
| pJB1806::*legC4* (pJB*legC4*)^b^ | *legC4* expression from endogenous promoter | Cm^R^ | [1] |
| *E. coli* expression |  |  |  |
| pGEX6P1 | For expression of GST-fusion proteins | Amp^R^ | GE Healthcare |
| pGEX::*psme1* | Expression of GST-PA28a | Amp^R^ | This study |
| pGEX::*legC4* | Expression of GST-LegC4 | Amp^R^ | This study |
| pGEX::*lgt1* | Expression of GST-Lgt1 | Amp^R^ | [2] |
| pT7HMT | Expression of His_6_-Myc fusion proteins | Kan^R^ | [5] |
| pT7HMT::*psme1* | Expression of His_6_-Myc-PA28a | Kan^R^ | This study |
| pT7HMT::*legC4* | Expression of His_6_-Myc-LegC4 | Kan^R^ | This study |
| pT7HMT::*lgt1* | Expression of His_6_-Myc-Lgt1 | Kan^R^ | [2] |
| Mammalian Expression |  |  |  |
| pcDNA 3FLAG 4/TO | For ectopic FLAG-fusion production | Amp^R^ | [6] |
| pcDNA::*3xflag-legC4* | Ectopic production of FLAG-LegC4 | Amp^R^ | This study |
| pEGFPC1 | Expression of GFP-fusion proteins | Kan^R^ | Clontech |
| pEGFPC1::*psme1* | Ectopic production of GFP-PA28α | Kan^R^ | This study |
| pCMV-3Tag-4a::*psme1*^c^ | Ectopic production of PA28α-Myc | Kan^R^ | This study |

a – gene expression induced with 1 mM IPTG

b – *legC4* expression from endogenous promoter

c – purchased from Genscript (Piscataway, New Jersey)

**Table C**. Oligonucleotide primers used in this study

| **Name** | **Sequence (5'🡪3')^a^** |
| --- | --- |
| LegC4BamHI-F3 | ATTGGATCCTTGATTCATTATGTATCCTTG |
| LegC4NotI-R | ATTGCGGCCGCTTATAGCTTAATATCAAAAG |
| Psme1Sal1-F | ATTGTCGACATGGCCACACTGAGGGTCCATCCC |
| Psme1BamHI-R  Psme1BamHI-F  Psme1NotI-R | ATTGGATCCTCAATAGATCATTCCCTTGGTTTC  ATTGGATCCATGGCCACACTGAGGGTCCATCCC  ATTGCGGCCGCATTCAATAGATCATTCCCTTGGTTTC |

a – restriction endonuclease cleavage sites are underlined.

**References**

1. Ngwaga T, Hydock AJ, Ganesan S, Shames SR. Potentiation of Cytokine-Mediated Restriction of *Legionella Intracellular* Replication by a Dot/Icm-Translocated Effector. *J Bacteriol.* 2019;201: e00853-19

2. Joseph AM, Pohl AE, Ball TJ, Abram TG, Johnson DK, Geisbrecht BV, et al. The Legionella pneumophila metaeffector Lpg2505 (MesI) regulates SidI-mediated translation inhibition and novel glycosyl hydrolase activity. Infect Immun. 2020;5: e00853-19

3. Folly-Klan M, Alix E, Stalder D, Ray P, Duarte LV, Delprato A, et al.  A novel membrane sensor controls the localization and ArfGEF activity of bacterial RalF. *Plos Pathog.* 2013;9: e1003747 (2013).

4. Bardill JP, Miller JL, Vogel JP. IcmS-dependent translocation of SdeA into macrophages by the Legionella pneumophila type IV secretion system. Mol Microbiol. 2005;56: 90-103

5. Geisbrecht BV, Bouyain S, Pop M. An optimized system for expression and puriﬁcation of secreted bacterial proteins. Protein Expr Purif. 2006;46: 23–32

6. A. Ingmundson, A. Delprato, D. G. Lambright, C. R. Roy, *Legionella pneumophila* proteins that regulate Rab1 membrane cycling. *Nature.* 2007;450: 365–369
