# Supplementary figures and images for: Correction: Effector-mediated subversion of proteasome activator (PA)28αβ enhances host defense against Legionella pneumophila under inflammatory and oxidative stress conditions
Source: PLoS Pathog. 2023 Aug 22;19(8):e1011604. doi: 10.1371/journal.ppat.1011604 (PMC10443833; doi:10.1371/journal.ppat.1011604)

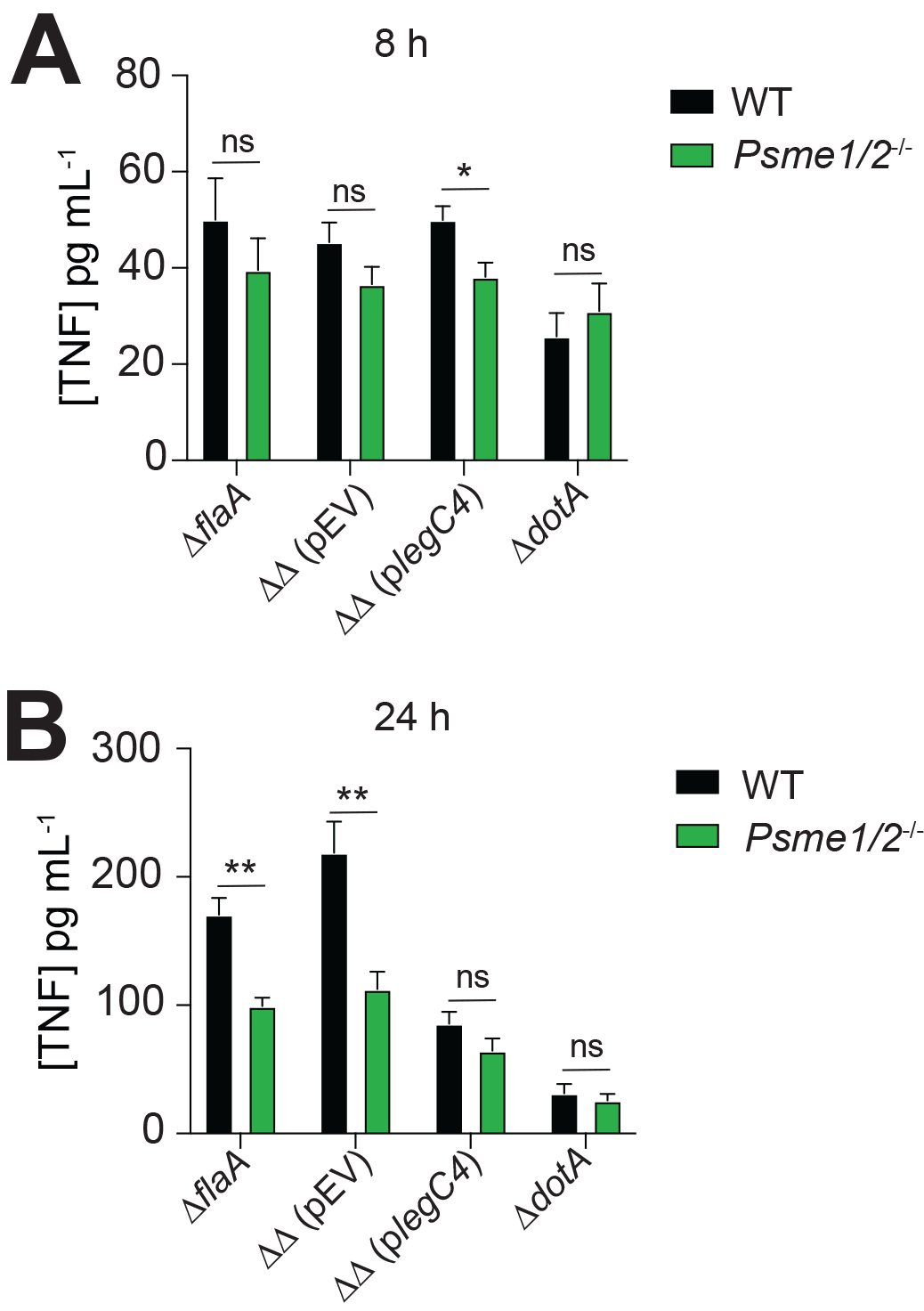

Supplement: S1 Fig — TNF WT or Psme1/2-/- BMDMs infected for (A) 8 h or (B) 24 h with L. pneumophila ΔflaA, ΔflaAΔlegC4 (pEV), ΔflaAΔlegC4 (plegC4), or the avirulent ΔdotA control at a multiplicity of infection of 10. Plasmid expression of legC4 was induced with 1 mM IPTG. Data shown are mean ± s.d. on samples in triplicates for a single experiment and are representative of results from three independent experiments. Asterisks denote statistical significance by two-way ANOVA (*P<0.05; **P<0.05). ns; not significant. (TIF) [file ppat.1011604.s001.tif]
